# Parental Educational Anxiety during Children’s Transition to Primary School in China

**DOI:** 10.3390/ijerph192315479

**Published:** 2022-11-22

**Authors:** Qianqian Liu, Xiumin Hong, Mei Wang

**Affiliations:** Faculty of Education, Beijing Normal University, Beijing 100875, China

**Keywords:** Chinese parents, parental educational anxiety, transition, preschool, primary school

## Abstract

Children’s transition from preschool to primary school has long been recognized as a particularly challenging period that can induce parental educational anxiety. As a true portraiture of Chinese parenting, educational anxiety during this transition has attracted considerable attention, given its detrimental effects on children’s development and education. This study was aimed at identifying the characteristics of parental educational anxiety and its influencing factors during children’s transition to primary school in China. We examined parental educational anxiety and parental educational experience through a questionnaire survey. The sample comprised 26,295 families with children in grade one distributed across nine cities in five provinces. The results indicate that (1) Chinese parents experience some degree of educational anxiety during their children’s transition to primary school and are more worried about their children’s learning and social adaptation processes than about how the curriculum is taught; (2) scores for parental educational anxiety varied significantly across different regions and urban/rural areas; (3) family socioeconomic status, parents’ educational concepts, preschools’ entrance preparation work, and primary school enrollment adaptation work are all important factors influencing parental educational anxiety during this transition. Our findings highlight the prevalence of parental educational anxiety in China and the need for effective measures to facilitate a smooth transition.

## 1. Introduction

The transition to primary school is a pivotal experience for young children, given its potential long-term impacts on school performance [1,2]. Within the literature, increasing attention has been focused on the family as a key contextual factor that strongly influences a child’s transition experience from kindergarten to primary school [3,4]. Families are also aware of the importance of being involved in their children’s transition process [5]. In addition to inducing excitement associated with the achievement of a major developmental milestone, entry into primary school can cause high levels of stress for children and their families because of discontinuities in the educational settings of preschools and primary schools [6,7].

Many parents develop anxiety during the period of their children’s transition between schools. Moving to primary school is inherently challenging for young children, and this challenge can be exacerbated by parental educational anxiety. Therefore, reducing parental educational anxiety during the transition period is necessary to facilitate a smooth transition for the children. In China, parental educational anxiety has always been a prominent social issue that has attracted the attention of the government, families, and schools. This study was aimed at investigating the characteristics and influencing factors of parental educational anxiety during the transition period in a Chinese cultural context.

### 1.1. Transition to Primary School in China

Differing from the preschool and primary school systems in other countries, such as the United States [8] and the United Kingdom [9], China’s preschool education targets children aged 3–6 years [10], whereas primary education targets children above 6 years. In other words, Chinese children attend primary school after graduating from preschool. In China, the transition from preschool to primary school is challenging for children and their families as well as for preschools and primary schools [11]. Moreover, this challenge is a longstanding one that is mainly attributable to differences in the learning styles, content, and objectives of primary schools and preschools. For example, preschool children learn through play; they explore and acquire knowledge during free play [12]. In contrast, primary school students learn by sitting upright in the classroom and listening quietly to the teacher [13].

The challenges entailed in the transition from preschool to primary school have attracted the attention of the Chinese Ministry of Education. The National Preschool Education Publicity Months organized in 2016, 2019, and 2022 focused on the transition from preschool to primary school [14,15,16]. However, parents who worry about their children’s post-enrollment adaptation also experience educational anxiety. In 2021, the Chinese Ministry of Education issued *Guidelines on the Vigorous Promotion of the Scientific Connection Between Preschools and Primary Schools* [17], which includes alleviation of parental educational anxiety during the transition to primary school. The Chinese Ministry of Education is committed to alleviating parental educational anxiety, which is a challenging task requiring long-term efforts. The question that arises and requires investigation is what are the characteristics and influencing factors of Chinese parental educational anxiety?

### 1.2. Parental Educational Anxiety during Children’s Transition to Primary School

The initiatives of educational policy makers, teachers, and families across the globe are directed toward the goal of having children enter primary school ready to succeed [18,19]. This emphasis on ensuring that children enter school ready to learn is partly attributable to empirical research findings that demonstrate a correlation between children’s readiness to succeed when they enter school and their later success in school and in life [18,20]. Positive transitions to formal schooling (primary/elementary school) are acknowledged to contribute to children’s subsequent educational achievements [21,22,23].

The transition to primary school not only impacts children but may also affect their parents and other family members [24]. Parents can have concerns and anxieties about various aspects of their children’s development during the transition period. Parents of preschool children experience anxiety relating to fear of the unknown associated with the change and the impending move of their children to a new environment. Studies have highlighted their awareness of the fact that their own disquietude can be transmitted to their children [5]. Parental educational anxiety not only pertains to academic issues but also to apprehension about their children’s ability to adapt socially and more generally in life. Parents may have significant concerns, including those related to their children’s behavior and academic skills [25]. One study found that 27.9% of parents expressed serious apprehension regarding their children’s transition from preschool to primary school, with sociobehavioral concerns reported most frequently by respondents [24]. Another study found that the top five concerns raised by caregivers of children transitioning from preschool were attending a new school, complying with/following directions, behavioral problems, academic skills, and getting along with peers [25].

Parental educational anxiety has thus become an important issue within family education in China [26]. In 2018, the results of a survey of 3205 parents across the country revealed the anxiety index score of Chinese parents as a whole. Specifically, they revealed that Chinese parents are in a state of anxiety, especially during their children’s early childhood and when they are in primary school [27]. Importantly, emerging research suggests that parents’ own anxiety may influence their engagement with their children’s education [28]. In addition, parental educational anxiety is viewed as a factor that generates stress in children [5]. Thus, the children of parents who express relatively more concern reportedly display more signs of anxiety [29]. Evidently, parental educational anxiety is detrimental to the children’s development and the family environment. Therefore, we focused on parental educational anxiety, with the aim of identifying ways of alleviating parental educational anxiety during the transition to primary school.

### 1.3. Factors Influencing Parental Educational Anxiety concerning Children’s Transition to School

Multiple factors contributing to parental educational anxiety are becoming increasingly apparent. For example, parental educational anxiety is related to parents’ own educational concepts. In one study, preschool teachers noted that parents who experienced educational anxiety relating to the transition tended to imagine their children in a highly demanding environment during their first year of school [5]. The gap in parental expectations has a significant positive effect on parental educational anxiety, with a wider gap corresponding to higher levels of parents’ educational anxiety [26]. Parental concerns regarding their children’s readiness appear to be linked to their awareness that their children would not fulfill and/or meet academic and social expectations in their new environments [18].

Previous studies have found that factors such as annual family income have a significant impact on parental educational anxiety [26]. One study reported that all of the interviewed parents expressed some anxiety relating to the transition [30]. These anxieties were greatly exacerbated when parents perceived that they had little or no choice because of economic pressures. Parental educational anxiety continuously directs the family’s cash flow and the children’s energy and outputs. Consequently, the phenomenon of involution of basic education deepens [31]. The pressure exerted on children to learn and parents’ anxiety about future social status are interwoven. The continued proliferation of off-campus training perpetually drains students’ energy and generates never-ending demands on parents relating to their tuition.

In addition to family-related factors, the preparatory work done by preschools and primary schools regarding transition connection also affects parental educational anxiety. One study reported that parents expressed concerns about their children’s transition and wanted to play an active role in planning the transition [25]. Consistent routines forged by parents and preschools are pivotal in influencing parents’ feelings about the transition. A study of Finnish parents found that mothers introduced some changes to their everyday life at home that were similar to the assumed routines at an early education institution to support their children’s transition [30]. More communication between families and institutions as well as the teachers’ professionalism allay parents’ anxiety. In particular, across countries, effective dialogues between parents and teachers are viewed as an important source of comfort and clarity [30].

### 1.4. The Current Study

An understanding of parental educational anxiety and its influencing factors during children’s transition to primary school can facilitate the development of more targeted educational interventions. As discussed above, the educational anxiety of Chinese parents during their children’s transition to primary school has become a hot social issue that has attracted the attention of China’s educational authorities. In light of this context, this study had two main objectives: (1) to understand the characteristics of parental educational anxiety during children’s transition to primary school, and (2) to identify the influencing factors of parental educational anxiety during the transition to primary school.

## 2. Methods

### 2.1. Participants

The participants in this study were respondents to a national survey conducted in China. They comprised 26,295 families with children in grade one at the time of their recruitment and were distributed in nine cities across five provinces. In this study, the sample was geographically representative, covering the eastern (32.0%), central (36.8%), and western (31.2%) regions of China. Within the sample, 19,830 respondents (75.4%) were mothers, 5461 respondents (20.8%) were fathers, and 1004 respondents (3.8%) were grandparents. The mean age of the parents was 33.53 years old (*SD* = 4.28). Of the children, 14,509 (55.2%) were boys and 11,786 (44.8%) were girls. Of these families, 13,360 (50.8%) were from cities, and 12,935 (49.2%) were from rural areas. According to information obtained from the National Bureau of Statistics of China in 2021 [32], most of the families in this sample were low- to middle-income households. The parents’ educational accomplishments were as follows: 69.5% had completed senior high school or below, 17.2% had completed up to 3 years of college, and 13.3% had completed 4 or more years of university.

### 2.2. Measurements

#### 2.2.1. Demographic Questionnaire

The demographic questionnaire comprised items pertaining to the parents’ education attainments, family incomes (estimated gross monthly income), and occupations. Education attainment was measured using a 3-point scale (1 = senior high school or below, 2 = up to 3 years of college, 3 = 4 or more years of university). Family income was measured using a 4-point scale (1 = under CNY 5000, 2 = CNY 5001–10,000, 3 = CNY 10,001–15,000, 4 = over CNY 15,000). According to Lu [33], occupational levels in China can be categorized into nine groups. Accordingly, the parental occupations were assigned a number ranging from 1 to 9, with higher numbers indicating greater occupational prestige. Family incomes, parental education, and occupations were standardized (Z-score), and family socioeconomic status (i.e., SES) was identified as an observed construct using five standard scores. Furthermore, data on the geographical regions (eastern, central, and western regions) and children’s characteristics were recorded.

#### 2.2.2. Parental Educational Anxiety Questionnaire

This questionnaire was designed for the current study to investigate parental educational anxiety during children’s transition to primary school. The survey items were derived from existing studies [25]. The questionnaire comprised 12 items covering three dimensions: four items on anxiety about children’s learning (e.g., “I worry about my child’s learning foundation”), four items on anxiety about children’s social adaptation (e.g., “I’m worried that my child won’t get along with their classmates”), and four items on anxiety about how the curriculum is taught (e.g., “I’m worried that the way primary school teachers teach is not suitable for children”). Parents responded to the items using a 5-point Likert scale ranging from 1 (*strongly disagree*) to 5 (*strongly agree*).

To verify the scale’s reliability and validity in the Chinese context, we performed a correlation analysis and a Cronbach’s analysis and tested the structural validity of the scale. The results of the correlation analysis indicate that the correlation matrix between the parental educational anxiety questionnaire and its three subdimensions ranged between 0.93 and 0.95 (*p* < 0.001). Cronbach’s alpha values for the three dimensions range between 0.88 and 0.93. The results of the confirmatory factor analysis (CFA) confirm that the three-factor model structure adequately fitted the data (*χ*^2^*/df* = 2.578, *p* < 0.001, comparative fit index (CFI) = 0.964, Tucker–Lewis index (TLI) = 0.953, and root mean square error of approximation (RMSEA) = 0.064 with a 90% confidence interval (CI, 0.062, 0.066)), which indicated a good fit.

#### 2.2.3. Parental Education Experience Questionnaire

In line with the policies of the Chinese Ministry of Education [17], we designed the parental education experience questionnaire, targeting children’s transition to primary school. The questionnaire comprised three parts: 15 items on parents’ educational concepts during their children’s transition to primary school (e.g., “I worry about my child’s learning foundation”), 3 items on parents’ perceptions of how well preschools prepared their children for school entrance (e.g., “The child’s preschool has done an adequate job in preparing them for primary school”), and 3 items on parents’ perceptions of the primary school’s efforts to facilitate their children’s post-enrollment adaptation (e.g., “I am satisfied with the efforts of my child’s primary school to help them to adapt after enrollment”). Parents responded to the items using a 5-point Likert scale ranging from 1 (*strongly disagree*) to 5 (*strongly agree*).

### 2.3. Procedures

This study was conducted with the support of the Ministry of Education and local education authorities. First, we randomly selected six provinces to represent Eastern, Central, and Western China. These provinces were Tianjin and Shandong in Eastern China, Henan and Jiangxi in Central China, and Guizhou in Western China. Next, we randomly selected 1–2 cities according to the list of cities in the province. As a third step, based on the principle of stratified sampling and convenient sampling, this study sent invitations to both urban and rural primary schools that we could contact. The list of primary schools was provided by local education authorities. After obtaining permission from the respective primary schools to conduct the study, we selected families according to the following criteria: (1) children without special needs, (2) children in grade one, and (3) two-parent families. To avoid the subject effect, we emphasized in our invitations to elementary schools and families that our study was anonymous, meaning that we would not collect the names of the primary schools, families, or children. After we had provided the parents with information about the research objectives and assured them that the information collected would be used solely for research purposes, they provided their consent to participate in the study. We sent our e-questionnaires containing the instructions to the parents. The parents received a separate e-questionnaire that they could fill out using a mobile phone or computer. We conducted a pilot study, which indicated that all the items could be completed within approximately 10 min. Therefore, in the main study, we set the time range for responding to the questionnaire items at 8–15 min. If the time taken to complete the questionnaire was outside this range, the questionnaire was rejected.

### 2.4. Data Analysis

We obtained descriptive statistics and performed internal consistency analysis using SPSS version 22. We performed CFA and latent profile analysis using Mplus version 7.4. The robust maximum likelihood estimator was used for the CFA to verify the three-factor structure of parental educational anxiety using the item scores in the current sample. Commonly used fit indices were employed, namely RMSEA, CFI, and standardized root mean square residual (SRMR) [34].

We performed latent profile analysis (LPA) to identify homogeneous parental educational anxiety groups (latent profiles). We also compared several fitting indexes among the models to determine the optimal models. Their values were obtained for the Akaike information criterion (AIC), the Bayesian information criterion (BIC), the sample size-adjusted Bayesian information criterion (SSA-BIC), entropy, and the Lo–Mendell–Rubin test (LMR) models. The entropy values were used to evaluate the accuracy of the model classification. Approximation of the entropy values closer to 1 reflected greater accuracy of the model classification. Entropy values ≥ 0.80 indicated that the accuracy of the classification was over 90% [35]. Moreover, a significant *p*-value of the LMR indicated that the k classes model was significantly better than the k−1 classes model [36].

## 3. Results

### 3.1. Overall Parental Educational Anxiety during the Transition to Primary School

Our results indicate that parental educational anxiety concerning children’s learning (*M* = 3.17, *SD* = 0.92) was slightly higher than that concerning their social adaptation (*M* = 3.09, *SD* = 0.91). However, the level of anxiety regarding the teaching of the curriculum was relatively low (*M* = 2.03, *SD* = 0.71). Higher mean scores were recorded for 3 of the 12 items, indicating relatively high anxiety levels relating to studies and post-enrollment adaptation during the transition to primary school. These three items were “I’m worried that the child can’t communicate well with the teacher” (*M* = 3.41, *SD* = 1.19), “I’m worried about my child not listening in class” (*M* = 3.73, *SD* = 1.18), and “I worry about my child’s poor academic foundation” (*M* = 3.35, *SD* = 1.18).

### 3.2. Latent Class Analysis of Parental Educational Anxiety during the Transition to School in China

To explore the potential characteristics of parental educational anxiety during children’s transition to school in China, we conducted an LPA. As a person-centered classification method, LPA was found to be superior to cluster analysis for analyzing covariates and complex variable relationships. Table 1 shows the LPA model fit indices for models with one to six profiles within the overall sample. Of the six models, two, namely LMR yielded significant values. The values of the AIC, BIC, and SSA-BIC indicate that the six-class solution performed slightly better than the others. However, the four-class model had the highest entropy value, which implies a more accurate classification. All indices show that the four-class solution was the best solution. We therefore selected this model as the optimal model.

**Figure 1:**
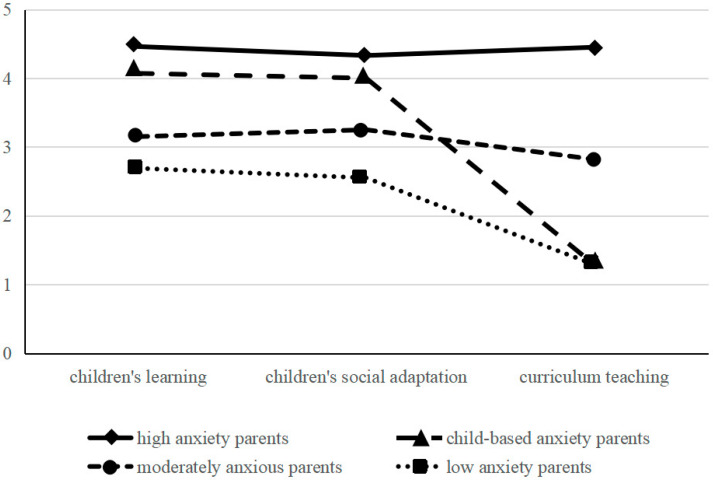
Latent classes of parental educational anxiety.

Figure 1 presents a graphical depiction of the four-class solution. Class 1 (8.8% of participants) evidences high scores for the three dimensions, with a mean score between 4.33 and 4.46. Parents in this class were therefore categorized as highly anxious parents. Class 2 respondents (26.8% of the total) scored higher for anxiety related to their children’s learning (*M* = 4.07) and social adaptation (*M* = 4.00) than for anxiety related to teaching the curriculum (*M* = 1.29). They were categorized as parents with child-focused anxiety. Class 3 parents (16.9% of the participants) were categorized as moderately anxious parents, given mid-level scores (ranging between 2.82 and 3.25) obtained for all three dimensions. Class 4 parents (47.5% of the participants) attained low scores (between 1.29 and 2.69) for all three dimensions and were categorized as parents with low anxiety levels.

### 3.3. Differences in Parental Educational Anxiety Scores during Children’s Transition to Primary School

As shown in Table 2, there were significant differences in parental educational anxiety scores among regions (*F* = 248.59 ***, *p* < 0.001; *F* = 36.90 ***, *p* < 0.001; *F* = 120.36 ***, *p* < 0.001). Parents in Western China had significantly higher scores than those in Eastern and Central China for anxiety relating to the three dimensions of children’s learning, social adaptation, and teaching the curriculum. Parents in Eastern China had significantly lower anxiety scores than parents in the two other regions for all three dimensions. In addition, the results of the independent sample *t*-test indicate that the scores for educational anxiety among urban parents were significantly lower than those for rural parents (*t* = −22.94 ***, *p* < 0.001; *t* = −10.97 ***, *p* < 0.001; *t* = −4.26 ***^,^ *p* < 0.001).

### 3.4. Factors Influencing Parental Educational Anxiety during Children’s Transition to Primary School in China

The three dimensions of parental educational anxiety were significantly correlated with family SES, parental education concepts, preschools’ entrance preparation work, and primary schools’ efforts to facilitate children’s post-enrollment adaptation. The values of the correlation coefficients ranged between 0.28 and 0.45. We conducted two-step hierarchical regression analyses using the enter method to predict scores for children’s learning and social adaptation and for teaching the curriculum. The geographical regions (East, Central, and West China) and areas (urban and rural) were significantly related to some of the study variables and were controlled during the first step. In the second step, family SES, parental education concept, preschools’ entrance preparation work, and primary schools’ efforts to facilitate post-enrollment adaptation were entered. The results of these hierarchical regression analyses show that family SES, parental education concepts, preschools’ entrance preparation work, and primary schools’ efforts to facilitate children’s post-enrollment adaptation were significant factors influencing parental educational anxiety in China. As Table 3 shows, the significant influencing factors were all negatively associated with the three dimensions of parental educational anxiety. Of these factors, parental educational concepts had the greatest influence on parental educational anxiety relating to the three dimensions: children’s learning and social adaptation and teaching the curriculum.

After controlling for family SES, we found that preschools’ entrance preparation work, primary schools’ efforts to facilitate post-enrollment adaptation, and parental education concepts could still explain the negative association with parental educational anxiety. The value of the standardized regression coefficient was 0.34. In sum, our findings indicate that parental education concepts are the key factors affecting parental educational anxiety.

## 4. Discussion

We found that educational anxiety is prevalent among parents and that the level of their psychological burden is high. Parental educational anxiety experienced within families stems from the education system and encompasses aspects such as the quality of education and the social milieu in which education takes place. Educational anxiety does not just reflect parents’ educational mindset; it is also indicative of a negative social milieu in which children learn and develop. This milieu reflects the parents’ own flawed educational mindset as well as the deficits within the educational system. It is imperative to relieve parents’ educational anxiety, but doing so requires comprehensive and long-term efforts given the complexity of this issue. Our findings indicate that family SES, parents’ educational concepts, preschools’ entrance preparation work, and the efforts of primary schools to facilitate post-enrollment adaptation are all important factors influencing parental educational anxiety during children’s transition to primary school. They can contribute to the development of targeted interventions to educate parents and alleviate their educational anxiety.

### 4.1. Parents’ Educational Anxiety during the Transition to Primary School

As the primary influencers in their children’s lives, parents are not only involved in their children’s transition from preschool to primary school but they also worry about whether their children will make the transition smoothly. Our findings indicate that Chinese parents experience a certain degree of educational anxiety during their children’s transition to primary school. More than half of the parents in the study were classified as having some level of educational anxiety (high, child-focused, or moderate). This anxiety may occur because the transition from preschool to primary school is a challenging event. Evidently, concern about whether children will make this transition smoothly is not confined to Chinese parents. A study on American parents found that parents had social as well as academic concerns relating to their young children, fearing that they would not be able to meet the academic and social demands in the new environment [18].

A notable finding of our study was that the parents worried more about their children’s learning and social adaptation than about how the curriculum was taught. More than a quarter of them were concerned about their children. That is, 26.8% of parents were categorized as having child-focused anxiety, which may be related to China’s Confucian culture. It is widely acknowledged that parents in East Asian countries, in particular, greatly value their children’s achievements, notably their academic success [37]. “One chance” national exams are prevalent in China, contributing to merit-based social mobility. The desire for social mobility may therefore induce a state of constant anxiety in parents regarding their children’s ability to overcome academic competition [37]. In addition, respecting teachers and attaching importance to education are traditional Chinese views [38] that still endure in the present. Accordingly, parents influenced by the view of respecting teachers may be more relaxed about their teaching method.

### 4.2. Regional Differences in Parental Educational Anxiety during Children’s Transition to Primary School

Our findings indicate that levels of parental educational anxiety were higher in Western China than in the other regions and in rural versus urban areas. A possible reason is the contrast between parents’ increased educational awareness and the low educational quality in Western China and in rural areas. With more parents from the post-80s and post-90s generations in Central and Western China and in rural areas, parents’ knowledge has generally improved, with parents paying more attention to their children’s education. Given their overall higher educational expectations, their expectations of high-quality and balanced education for their children have also been strengthened [39,40]. However, despite the overall level of development of Chinese education, which now occupies the middle and top ranks globally, the supply of high-quality education resources remains inadequate and unbalanced. Marked differences in the quality of education among regions, urban and rural areas, and schools persist [41]. Unbalanced resource allocation within preschool education in China is a prominent issue, which constrains the sustainable development of preschool education [42]. For example, one study found that imbalances relating to the distribution of educational resources and the quality of education, unqualified teachers, and unreasonable curricula in preschools as well as substandard private preschools persist in rural areas [43].

There is a huge gap between parents’ aspirations for high-quality education resources in Western China and rural areas and the reality of imbalances in the supply of education resources. Consequently, parents in Western China and rural areas are not satisfied with the educational status of their children, which induces excessive worry, dissatisfaction, and concern about their children’s learning and how curricula are taught, leading to high levels of educational anxiety. Thus, the uneven allocation of basic education resources has become a fundamental source of high levels of educational anxiety among parents in Central and Western China and in rural areas.

### 4.3. Factors Influencing Parental Educational Anxiety during the Transition to Primary School

We found that family SES, parents’ educational concepts, preschools’ entrance preparation work, and primary schools’ efforts to facilitate post-enrollment adaptation are all important factors influencing parental educational anxiety during children’s transition to primary school. It is noteworthy that parental education concepts had the greatest influence on parents’ educational anxiety. This finding may be linked to China’s educational culture. Parental educational anxiety has historical roots, although it is now more prominent. China has always valued the social function of education, considering education as an important tool for effecting class change [44,45]. Influenced by the traditional adage that “education changes destiny”, parents’ expectations of their children being squeezed into increasingly narrow upward channels have acquired more urgency, with a greater focus on the role of education, which they view as the only “low-cost” tool for changing “destiny”. Under the logic of utilitarianism, it has become common for parents to worry excessively about the results and processes of their children’s education. However, utilitarianism deviates from the essence of education, restricting students’ overall development, and it damages the education process. Fundamentally, this phenomenon stems from unscientific parental education concepts and a lack of an objective understanding of children’s development and educational activities.

Our findings show that family SES was negatively associated with parental educational anxiety, which may be related to the cost of educating children during the transition. Family SES determines the ability to provide children with school supplies and enable them to attend interest classes and training courses [41]. Parents with low SES may worry that their children will fall behind, given the deficit in school supplies, interest classes, and training courses. In addition, the higher level of parental educational anxiety among low SES parents may be related to their perceptions of their own educational inadequacy [39]. Parents with low SES may be aware that they lack the educational competence to tutor their children, who consequently fall behind.

An important part of preschool education entails preparing children for school in a scientific manner. For example, the *Work Regulations for Preschools* point out that preschools and primary schools should maintain close contact, cooperate with each other, and attend to their educational connections [46]. In view of the importance of preschools’ entrance preparation work, the Ministry of Education issued *Key Points of Education Guidance for Preschool Entrance Preparation*, which provides specific guidance on this subject [47]. Adequate preparation for entrance into primary school is not only an important basis for young children to adapt well to primary education [18,22,24]; it is also a way to alleviate parental educational anxiety. The results of this study show that preschools’ entrance preparation work could indeed alleviate families’ educational anxiety.

We also found that primary schools’ post-enrollment adaptation work affects parental educational anxiety. A likely reason is that to a certain extent, these efforts influence children’s transition and post-enrollment adjustment during their first school year [4,48]. Whether or not children successfully adapt to primary school education during the initial stage determines their attitudes and emotions toward their future school lives to some extent and affects their future academic performance and development. Therefore, the work of primary schools in China and around the world in facilitating post-enrollment adaptation is essential [9,11,49,50].

### 4.4. Practical Implications

Our findings have important practical implications for policy makers, educators, and parents. First, they can enhance awareness among policy makers of the gravity of severe parental educational anxiety in China and of regional and urban variations. Policy makers should continue to focus on parental educational anxiety and direct high-quality educational resources toward Western China and rural areas [26]. For instance, the government could establish regulations to increase opportunities for families in need to obtain more community resources for cultural activities (e.g., museum visiting). It could also expand resources such as famous public teachers’ classes through online platforms [31], which could address parents’ concerns regarding their children’s learning and social adaptation. Second, educators should take effective measures to strengthen connections between preschools and primary schools. A previous study found that providing only financial resources to economically weak families is not an effective solution in the absence of parenting knowledge [51]. Therefore, educators should augment preschools’ entrance preparation work and primary schools’ post-enrollment adaptation work by providing scientific guidance on parenting. They should also encourage parents’ involvement during their children’s transition to primary school, which could reduce their educational anxiety. In addition, parents should develop scientific educational concepts and the concept of becoming successful [26]. They should also recognize and actively embrace the important role of parental involvement in children’s development and education.

## 5. Limitations of the Study

This study has several limitations. First, because of COVID-19 restrictions, we used a digital questionnaire to collect the participants’ information. Future studies could entail interviews, questionnaires, and other methods to obtain more objective and comprehensive data. Second, the participants were mostly mothers, with considerably fewer fathers, necessitating cautious interpretation of the findings for parental educational anxiety. The levels of involvement of fathers and mothers in parenting differ. Therefore, researchers could examine the educational anxiety of fathers and mothers separately. Third, the influencing factors included in this study on parental educational anxiety were limited. There may be other factors influencing parental educational anxiety, such as the level of children’s development and the degree of parental involvement, which indicate future research directions. Fourth, although we excluded children with special needs from the sample selection and ignored the impact of children’s learning disorders on parental educational anxiety. It is suggested that future studies could clarify the screening criteria at the stage of sample selection or consider learning disorders as an influential factor in the study.

## Figures and Tables

**Table 1:** Comparison of the fit index of different latent class models.

Model	AIC	BIC	SSA-BIC	Entropy	LMR *p*	Latent Class Probability
Class 1	90,359.152	90,403.536	90,384.468	-	-	-
Class 2	84,532.067	84,606.040	84,574.261	0.903	<0.001	0.888/0.112
Class 3	79,964.273	80,067.835	80,023.345	0.916	<0.001	0.025/0.170/0.805
Class 4	78,023.913	78,157.065	78,099.863	0.936	<0.001	0.088/0.268/0.169/0.475
Class 5	74,369.818	74,532.559	74,462.645	0.857	<0.001	0.584/0.131/0.181/0.084/0.020
Class 6	72,920.608	73,112.939	73,030.314	0.890	<0.001	0.014/0.018/0.575/0.180/0.072/0.141

Notes. AIC = Akaike information criteria; BIC = Bayesian information criteria; SSA-BIC = sample size-adjusted BIC; LMR *p* = *p*-value of the Lo–Mendell–Rubin test.

**Table 2:** Descriptive statistics for parental educational anxiety in relation to different background variables.

		Parental Educational Anxiety of Children’s Learning	Parental Educational Anxiety of Children’s Social Adaptation	Parental Educational Anxiety of Curriculum Teaching
Geographical region	Eastern region (*n* = 8396)	3.03 (0.97)	3.05 (0.93)	1.88 (1.04)
	Central region *(n* = 10,369)	3.15 (0.93)	3.08 (0.90)	2.12 (1.08)
	Western region (*n* = 7530)	3.36 (0.91)	3.17 (0.92)	2.08 (1.08)
*F*		248.59 ***	36.90 ***	120.36 ***
Living area	Urban area (*n* = 13,360)	3.04 (0.94)	3.03 (0.90)	2.00 (1.05)
	Rural area (*n* = 12,935)	3.30 (0.93)	3.16 (0.92)	2.05 (1.96)
*t*		−22.94 ***	−10.97 ***	−4.26 ***

Notes. *** p < 0.001.

**Table 3:** Factors influencing parental educational anxiety.

Variable	Parental Educational Anxiety of Children’s Learning	Parental Educational Anxiety of Children’s Social Adaptation	Parental Educational Anxiety of Curriculum Teaching
Family SES	−0.12 ***	−0.11 ***	−0.08 ***
Parental education concept	−0.18 ***	−0.14 ***	−0.29 ***
Preschool entrance preparation work	−0.10 ***	−0.07 ***	−0.08 ***
Primary school enrollment adaptation work	−0.11 ***	−0.10 ***	−0.17 ***
	Δ*R*^2^ *=* 0.246, *F =* 420.61	Δ*R*^2^ *=* 0.146, *F =* 141.61	Δ*R*^2^ *=* 0.347, *F =* 890.46

Notes. ^***^ p < 0.001. Δ*R*^2^: The adjusted *R*^2^.
